# Twin Similarity for Neighborhood, Geographic Mobility, and Health Outcomes in Late Adulthood

**DOI:** 10.1093/geroni/igab046.948

**Published:** 2021-12-17

**Authors:** Deborah Finkel, Ida Karlsson, Malin Ericsson, Tom Russ, Anna Dahl Aslan, Nancy L Pedersen

**Affiliations:** 1 Indiana University Southeast, New Albany, Indiana, United States; 2 Karolinska Institutet, Stockholm, Stockholms Lan, Sweden; 3 University of Edinburgh, Edinburgh, Scotland, United Kingdom; 4 Hälsohögskolan Skövde, Skövde, Vastra Gotaland, Sweden

## Abstract

Socioeconomic status (SES) is one of the most robust predictors of health. The source of SES-health associations is heavily debated; one approach is investigating neighborhood-level environmental characteristics. Challenges include selection effects and the possibility of reverse causation: people choose their neighborhoods. Longitudinal twin research can overcome these issues by assessing location choice over time as well as twin similarity; however, few existing twin studies have incorporated neighborhood-level data, and none of those focus on aging. Using longitudinal data from the Swedish Adoption/Twin Study of Aging, the current study examined the impact of location at various points in life. Location at birth and in 1993 were available for 972 participants. Birth years ranged from 1926 to 1948; mean age in 1993 was 54.55 (range = 35-67). Thirty-nine percent of the sample had moved to a different county between birth and midlife: individuals who moved had significantly higher parental SES and had achieved significantly higher education. Moreover, identical twin concordance for geographic mobility (77%) was significantly higher than fraternal twin concordance (65%), indicating a modest but significant genetic contribution. Geographic mobility did not impact identical twin similarity on a functional aging factor (corrected for age and education), but fraternal twins concordant for mobility were more similar than discordant twins, suggesting genetic contributions to mobility may also impact health. Ongoing retrieval of location information for twins born 1900-1925 and geocoding of location information available at 9 waves of data collection will allow for expanded investigation of the SES-health relationship at the neighborhood level.

